# From Professional Commitment to Continuity: How Resilience and Empowerment Reduce Turnover Intention Among Critical Care Nurses

**DOI:** 10.1155/jonm/4887342

**Published:** 2026-10-26

**Authors:** Sameer A. Alkubati, Ibrahim Alasqah, Nada Alqarawi, Wesam T. Almagharbeh, Heba E. El-Gazar, Mohamed A. Zoromba, Talal Al-Qalah, Basma Salameh, Mesheil Alalyani, Eddieson Pasay-an, Ahmed Hashem El-Monshed, Majid Ali Alotni, Shimmaa Mohamed Elsayed

**Affiliations:** ^1^ Department of Medical-Surgical Nursing College of Nursing University of Hail Hail Saudi Arabia uoh.edu.sa; ^2^ Department of Community, Psychiatric, and Mental Health Nursing College of Nursing Qassim University Buraydah 52571 Saudi Arabia qu.edu.sa; ^3^ Medical Surgical Nursing Department University of Tabuk Tabuk Saudi Arabia ut.edu.sa; ^4^ Nursing Administration Department Faculty of Nursing Port Said University Port Said Egypt psu.edu.eg; ^5^ College of Nursing Prince Sattam Bin Abdulaziz University Al-Kharj Saudi Arabia psau.edu.sa; ^6^ Psychiatric and Mental Health Nursing Department Faculty of Nursing Mansoura University Mansoura Egypt mans.edu.eg; ^7^ Faculty of Nursing Arab American University Jenin State of Palestine aauj.edu; ^8^ College of Nursing King Khalid University Abha Saudi Arabia kku.edu.sa; ^9^ Department of Nursing College of Health and Sport Sciences University of Bahrain Manama Bahrain uob.edu.bh; ^10^ Department of Medical-Surgical Nursing College of Nursing Qassim University Buraydah 52571 Saudi Arabia qu.edu.sa; ^11^ Critical Care and Emergency Nursing Department Faculty of Nursing Damanhour University Damanhour Egypt damanhour.edu.eg

**Keywords:** clinical practice, critical care nurses, intention to leave, professional commitment, psychological empowerment, resilience

## Abstract

**Aim:**

To examine the mediating effects of resilience and psychological empowerment on the relationship between professional commitment and intention to leave among critical care nurses (CCNs).

**Background:**

The intention of CCNs to leave their jobs is considered a primary problem faced by hospitals and healthcare executives worldwide. There is a need to analyze its relationship with professional commitment, resilience, and psychological empowerment.

**Design:**

A cross‐sectional study.

**Methods:**

This study was conducted among 430 CCNs from October 2024 to January 2025. Data were collected using a sociodemographic sheet, Psychological Empowerment Scale (PES), Intention to Leave Scale, Connor–Davidson Resilience Scale, and Nursing Professional Commitment Scale (NPCS). A path analysis was performed using AMOS 24.0 software.

**Results:**

Professional commitment demonstrated a significant total negative effect on intention to leave (*β* = −0.420, *p* = 0.005). Both resilience and psychological empowerment significantly mediated this relationship. The indirect effect through resilience was stronger (*β* = −0.071, *p* = 0.011) than that through psychological empowerment (*β* = −0.041, *p* = 0.004). Professional commitment was positively associated with both resilience (*β* = 0.208, *p* = 0.009) and psychological empowerment (*β* = 0.462, *p* = 0.009), while each mediator was negatively related to intention to leave (*β* = −0.343 and *β* = −0.091, respectively). The direct effect of professional commitment on intention to leave remained significant (*β* = −0.306, *p* = 0.005), indicating partial mediation.

**Conclusion:**

This study indicates that professional commitment, through fostering resilience and psychological empowerment, directly and indirectly lowers turnover intentions. These findings highlight the importance of enhancing nurses’ commitment, empowerment, and resilience in order to support workforce retention. Practical and administrative policy efforts that promote professional development, autonomy, and psychological support for nurses may help to reduce turnover rates in critical care settings.

**Implications for Nursing Management:**

Hospital administrators and nursing leaders should give top priority to initiatives that strengthen professional commitment**,** psychological empowerment, and resilience among CCNs, as these factors were found to directly and indirectly reduce intention to leave. Supportive leadership techniques, resilience‐building seminars, structured mentorship programs, and participatory decision‐making models may all help to reinforce nurses’ commitment to the profession. By incorporating these strategies into organizational policy, critical care settings can promote a stable nursing workforce, increase employee retention, decrease the desire to leave, and ultimately enhance the quality of patient care.

**Implications for Profession and/or Patient Care:**

Enhancing the psychological empowerment, resilience, and professional commitment of CCNs may successfully reduce their intention to leave and improve workforce stability and patient care quality. These results can be used by nursing administrators to create retention‐focused interventions, such as resilience training, empowerment initiatives, and mentorship, that promote nurses’ mental health and job happiness.

**Reporting Method:**

The STROBE checklist was used as a guideline.

**Patient or Public Contribution:**

No patient or public contribution.

**Impact Statements**
• What is known about the topic?
◦ High turnover intention among CCNs is a pivotal global concern, often linked to stress, burnout, and insufficient organizational support.
◦ Professional commitment, resilience, and psychological empowerment are independently recognized as factors that influence job satisfaction and retention among CCNs.
• What this paper adds?
◦ This study provides empirical evidence that resilience and psychological empowerment jointly mediate the relationship between professional commitment and intention to leave among CCNs.
◦ This highlights that fostering empowerment and resilience not only enhances CCNs’ professional commitment but also directly and indirectly decreases their intention to leave, offering a comprehensive model for workforce retention.

## 1. Introduction

Nursing serves as the foundation for healthcare systems and is considered the initial step of the connection between patients and the provision of healthcare services [1]. According to the World Health Organization (WHO), to meet the growing demand for healthcare, there may be a need for more than nine million nurses globally by 2030 [2]. Critical care nurses’ (CCNs) intention to leave their jobs is considered the primary problem faced by hospitals and healthcare executives worldwide. It significantly affects the stability, productivity, and performance of healthcare facilities [3, 4]. The intention to leave can be defined as the desire to resign and look for a new position or division, either internally (in the same organization) or externally (in another organization). It can also refer to quitting the profession entirely [3]. Nurses’ intention to leave was rated at 61.3% in Ethiopia [4] and 71.44% in Egypt [5].

Professional commitment can be defined as a sense of responsibility for the unique issues and challenges faced by a profession, loyalty, and the desire to remain in that profession [6]. Professional commitment is a multifaceted term that includes having a positive intrinsic value for work, wanting to stay in the field, believing in goals and values, and being willing to make an effort [7]. Professional commitment is believed to be one of the factors affecting nurses’ intention to leave [3]. Previous studies revealed a significant negative relationship between nurses’ professional commitment and intention to leave in Portugal [3] and Egypt [5].

Resilience is crucial for CCNs who are exposed to a variety of stressors from critically ill patients and their families, relationships with other staff members, and a complex work environment [8]. In addition to being essential for nurses’ personal health, resilience in nursing has a significant influence on patient care, and it is more likely that resilient CCNs will continue to perform well under pressure. Resilience can play a role in reducing CCNs’ intention to leave through their adaptation to stressful situations, specifically in intensive care units (ICUs) [9–11]. In a study conducted in Finland, the results indicated that higher resilience among nurses improved their quality of care and enhanced their job satisfaction, which ultimately reduced their intention to leave [12]. Another study in China revealed a positive correlation between nurses’ psychological resilience and their intention to stay (*r* = 0.388, *p* < 0.01) [13].

Psychological empowerment is another important factor affecting CCNs’ intention to leave. Employees who feel psychologically empowered take ownership and manipulate their workplace responsibilities and outcomes. In addition, they see their employment as worthwhile and meaningful, feel competent, and think they can have a big impact on the organization [14]. These empowerment characteristics meet employees’ psychological requirements, resulting in increased intrinsic motivation [14–16]. One study revealed that intention to leave was negatively correlated when employers were motivated internally or externally [17]. This confirms that genuinely motivated people are expected to stay in their employment and decrease their thinking about leaving.

## 2. Background

The research found that CCNs who strongly identify with their profession’s values are more likely to stay in high‐pressure critical care roles, partly because their commitment builds their resilience and sense of empowerment [18–20]. This study’s theoretical framework was based on complementary theories, including organizational commitment and conservation of resources (COR) theories. According to Meyer and Allen (1991), organizational commitment theory states that organizational commitment includes affective commitment (emotional attachment), continuance commitment (cost‐based attachment), and normative commitment (obligation‐based attachment) [21]. The concept of professional commitment may differ from that of organizational commitment, which focuses on attachment to a specific workplace rather than a broader profession. Deep commitment to one’s career, where the individual strongly identifies with their field, values independence, and embraces core values and goals. Essentially, the more someone is committed to their profession, the less likely they are to consider leaving it [20].

COR theory is a psychological framework that explains how people manage stress by focusing on the resources they have and want to maintain and strive to protect [22]. The theory posits that individuals who can fill and protect their emotional and psychological resources (e.g., resilience) are less prone to burnout and turnover [22, 23].

Recent studies have substantially advanced the understanding of nurses’ turnover intention by examining the roles of leader emotional intelligence, team culture, autonomous motivation, supervisor and coworker behaviors, resilience, work engagement [24–26], and nurses’ quality of life as important [27] organizational determinants of workforce retention. However, rather than looking at how individual psychological resources together explain the association between professional commitment and intention to leave, these studies have often examined these aspects either independently or within constrained explanatory models. Specifically, there is still little information on whether psychological empowerment and resilience work in conjunction as complimentary mediation processes between turnover intention and professional commitment among CCNs, who operate in extremely challenging clinical settings. Filling this gap is crucial because nursing managers may be able to create more all‐encompassing retention methods that enhance nurses’ adaptability and professional dedication while lowering turnover intention in critical care settings by identifying modifiable psychological factors.

Consequently, we aimed in this study to evaluate the mediating effect of resilience and psychological empowerment on the relationship between professional commitment and intention to leave among CCNs.

## 3. Aim of the Study

This study investigated the mediating effect of resilience and psychological empowerment on the relationship between professional commitment and intention to leave among CCNs.

### 3.1. Hypothesis development

#### 3.1.1. Professional Commitment and Intention to Leave

Organizational commitment theory states that people who have a strong emotional and normative bond with their area absorb its fundamental principles and responsibilities. This inherent professional identity serves as a psychological foundation for CCNs. When CCNs have a strong commitment to the nursing profession, they are more likely to see obstacles at work as part of their job rather than as grounds to quit, which immediately lessens their desire to leave. Therefore, the following hypothesis is proposed:

 Hypothesis 1: There is a negative relationship between CCNs’ professional commitment and intention to leave.

#### 3.1.2. Professional Commitment as a Source of Empowerment and Resilience

According to the COR theory, people work to develop and safeguard psychological resources to cope with stress. This is supported by organizational commitment theory, which contends that a solid professional identity acts as a fundamental “resource pool.” In addition to fostering an internal sense of control and competence (psychological empowerment) and developing the emotional capacity to overcome challenges (resilience), nurses who are passionately dedicated to their profession are more likely to take an active role with their work environment. Therefore, the following hypotheses are proposed:

 Hypothesis 2: There is a positive relationship between CCNs’ professional commitments and resilience.
 Hypothesis 3: There is a positive relationship between CCNs’ professional commitment and psychological empowerment.

#### 3.1.3. Protective Roles of Resilience and Empowerment

According to COR theory, psychological empowerment and resilience serve as essential protective mechanisms against occupational burnout. The high‐stress atmosphere of the ICU quickly drains emotional vitality. High resilience CCNs are able to adjust to various stimuli without suffering from extreme psychological fatigue. In a similar vein, nurses who are empowered and believe they have control over their work are less likely to experience overwhelm. As a result, these resources avoid the emotional exhaustion that usually leads to turnover intention. Therefore, the following hypotheses are proposed:

 Hypothesis 4: There is a negative relationship between CCNs’ resilience and intention to leave.
 Hypothesis 5: There is a negative relationship between CCNs’ psychological empowerment and intention to leave.

### 3.2. Mediating Mechanisms

Integrating previous hypotheses, we suggest a progressive protective mechanism. While professional commitment serves as the foundational motivator for nurses to stay in the field, CCNs’ day‐to‐day retention is dependent on how this commitment manifests itself in practice. We propose that professional commitment provides nurses with the psychological empowerment and resilience required to buffer the acute stressors of critical care, reducing their desire to leave. Therefore, the following hypotheses are proposed:

 Hypothesis 6: Resilience mediates the relationship between professional commitment and intention to leave.
 Hypothesis 7: Psychological empowerment mediates the relationship between professional commitment and intention to leave.

## 4. Subjects and Methods

### 4.1. Study Design and Setting

This cross‐sectional study was conducted between October 2024 and January 2025, in accordance with the Strengthening the Reporting of Observational Studies in Epidemiology (STROBE) statement. This study included 16 ICUs from five hospitals: Alexandria Main University Hospital, New Alexandria Main University Hospital in Smouha, Alexandria University Students Hospital, Al‐Mouwasat University Hospital, and Al‐Hadra University Hospital. These hospitals are affiliated with Alexandria University. The total bed capacity in the five hospitals was 155. The total number of beds in Alexandria Main University Hospital was 83 beds divided as follows: 11 beds in unit I, 12 beds in unit II divided into two halls (A, B), 12 beds in unit III divided into three halls (A, B, C), 6 beds in unit IV, 12 beds in unit V divided into three halls (A, B, C), 6 beds in coronary unit, 6 beds in cardiac unit, 6 beds in neurological unit, 4 beds in unit VI, and 8 beds in medical units. The New Alexandria Main University Hospital in Smouha has two units: a general ICU (10 beds) and a neurological unit (6 beds). The Alexandria University Students Hospital has two units (unit I has 10 beds, and unit II has 11 beds). Al Hadra University Hospital has two units (unit I has nine beds, and unit II has 11 beds). Al Mouwasat University Hospital had 15 beds (unit I had 9 beds, and unit II had 11 beds).

### 4.2. Population and Sample Size

This study involved all the CCNs in the intended ICUs. They were included in the study if they were working in one of the predetermined settings, had at least 1 year of experience in the ICUs where they were employed, and were directly caring for patients. The sample size was established using OpenEpi, with a minimum sample size of 391 based on the following criteria: 99% confidence level, 5% absolute precision, and a population size of 950. In total, 430 convenience samples were collected after the addition of 10%.

Registered nurses who were currently working in adult ICUs and had at least 1 year of clinical experience in an ICU were eligible to participate. The 1‐year ICU experience criterion was established to ensure that participants had adequate exposure to the critical care environment and its associated professional and organizational demands before completing the study questionnaires.

### 4.3. Data Collection Tools

CCNs’ demographic data, including age, gender (male or female), marital status (single, married, and divorced/widowed), level of education (diploma, bachelor, and master), extra work (yes and no), nursing experience (in years), nurse‐to‐patient ratio, and working ICU (general, coronary, cardiac, and neurological), were collected. Professional commitment, intention to leave, resilience, and psychological empowerment were assessed using validated psychometric instruments.

### 4.4. Psychological Empowerment Scale (PES)

A 12‐item scale was used to assess the participants’ perceptions of psychological empowerment in the workplace [28]. Each of the four subdimensions contained three items: meaning, competence, self‐determination, and impact. For instance, “My job activities are personally meaningful to me” (meaning), “I have considerable opportunity for independence and freedom in how to do my job” (self‐determination), “I am self‐assured about my capability to perform my job” (competence), and “I have a great deal of control over what happens in my department” (impact) [29]. Participants rated their level of agreement on a seven‐point Likert scale, with 1 indicating significant disagreement and 7 indicating strong agreement. The mean score ranged from 12 to 84, with a higher score indicating more psychological empowerment [28]. A panel of specialists (four clinical nurses and two faculty colleagues) examined the validity in a previous study [30]. Cronbach’s alpha revealed high reliability for all 12 scale items (*α* = 0.98), particularly for the competence (*α* = 0.94), meaning (*α* = 0.96), impact (*α* = 0.93), and self‐determination (*α* = 0.93) subscales [30]. In our study, the reliability of the instrument was 0.96, and for each dimension, it was 0.93, 0.92, 0.84, and 0.98, respectively.

### 4.5. Intention to Leave Scale

This scale was developed by Kumar and Govindarajo (2014), which includes 12 items grouped into three indicators, each with four items. The three indicators are as follows: (1) perceived alternative work opportunities (items 1, 2, 3, 4), (2) job expectations (items 5, 6, 7, 8), and (3) turnover intention (9, 10, 11, 12) [31]. For instance, “I have confidence in finding another job.” A five‐point Likert scale was used, with one representing strong disagreement and four indicating strong agreement. The scoring system ranged from 12 to 60 [31], with higher scores indicating a high intention to leave and vice versa. The original instrument‐development study established its psychometric properties. Furthermore, content, construct, and face validity were conducted on the subfactors and items generated in the instrument [31]. The instrument’s overall reliability coefficient yielded a Cronbach’s alpha of 0.70. The reliability of the three indicators was 0.812, 0.782, and 0.803, respectively [31]. In our study, the instrument’s overall reliability coefficient yielded a Cronbach’s alpha of 0.90. The three indicators accepted reliability as follows: 0.79, 0.76, and 0.72.

### 4.6. Connor–Davidson Resilience Scale

Connor and Davidson (2003) developed this scale to measure a person’s capacity to deal with adversity [32]. It is a unidimensional instrument with ten items explaining the various facets of resilience. The scale primarily functions as an assessment of resilience, encompassing questions related to flexibility (1 and 5), self‐efficacy (2, 4, and 9), emotional regulation (10), optimism (3, 6, and 8), and cognitive focus/attention maintenance under stress (7) [32]. For instance, “I adapt to change.” It is scored on a 5‐point Likert scale, where 0 indicates that it is never true and 4 implies that it is true virtually every time [32]. The overall score fell between 0 and 40, with higher scores indicating higher resilience. The CD‐RISC has been extensively used to assess resilience in healthcare populations, including nursing populations, and its psychometric properties have been demonstrated in previous research [32, 33]. Exploratory factor analysis supported a one‐factor construct, and Cronbach’s *α* of the scale was confirmed in a previous study, which was 0.85 [32]. In this study, Cronbach’s *α* was 0.96.

### 4.7. Nursing Professional Commitment Scale (NPCS)

This scale was developed by Lu et al. (2000) to assess nurses’ professional commitment [34]. The scale has three subdimensions (belief in aims and ideals, willingness to put forth effort, and sustaining professional membership) and 26 items. For instance, “I have stayed in the profession at personal will and interest.” A four‐point Likert‐type scale was used to score each question’s responses. Nine items of the scale are reversed (items 14, 15, 16, 17, 18, 19, 20, 21, and 25). The scale has scores ranging from 26 to 104. The subdimensions that yielded the lowest and highest scores were “willingness to make an effort” (13–52), “maintaining professional membership” (8–32), and “belief in goals and values” (5–20). Higher values indicate a greater degree of professional commitment [35]. The validity of the tool was confirmed in previous studies [34, 36]. Cronbach’s *α* coefficient of the scale in a previous study was 0.91 [35] and 0.98 in our study.

To confirm the instruments’ content validity and contextual suitability for the local population, a panel of five nursing experts (three academic faculty members and two senior critical care nursing directors) reviewed the original English questions first. The panel assessed the scales for clinical relevance and conceptual clarity within the Egyptian healthcare context. The experts concluded that the original English items were perfectly adequate and needed no adjustments. Prior to the primary data collection phase, a pilot study was conducted with a convenience sample of 43 CCNs from participating ICUs who met all inclusion criteria. The major goal of this pilot study was to assess the comprehensibility, clarity, and cultural acceptability of English‐language questionnaires, as well as estimate the time required to complete them. The pilot participants reported that the items and instructions were clear, unambiguous, and easy to understand in their original English version. As a result, no changes or cultural adaptations were required for any of the instruments. All questionnaires were administered in English, with no translations, linguistic adaptations, or instrument modifications. The questionnaires and accompanying instrument information are publicly available online from their original sources. As a result, no permissions for translation or cross‐cultural modification were required.

### 4.8. Data Collection

The process of gathering data was started in cooperation with the attending hospitals. CCNs were given self‐administered questionnaires to complete whenever it was convenient for them. All questionnaires were administered in English, as it serves as the official language for medical documentation, clinical communication, and professional practice within the intended ICUs of the participating hospitals. Therefore, no forward–backward translation or reconciliation procedure was required, and the original English versions of the validated instruments were used without translation or crosscultural adaptation. Because of this participant‐centered approach, nurses were able to answer honestly and deliberately without feeling pressured by the researcher. To ensure a comfortable and private environment for introspection, they could complete the questionnaires at home, during breaks, or after shifts in designated quiet places. In the end, this flexibility improved the quality and dependability of the data by accommodating their schedules. After determining the eligible participants, the researcher contacted them to explain the goals of the study, and informed consent was obtained from each participant. Questionnaires were sent with explicit instructions on how to complete all items to prevent missing data and submit them when consent was obtained. Completed questionnaires were sent back in sealed packages to ensure privacy. Upon receipt, the responsible researcher immediately screened the sealed packages to verify that the questionnaires were filled out completely. Questionnaires with missing or unengaged responses were excluded from the final dataset to guarantee the validity of the collected data. Frequent follow‐ups were carried out to maintain data integrity, resolve any participant queries regarding the forms, and promote timely submission.

### 4.9. Data Analysis

Data were analyzed using the IBM SPSS Statistics software, Version 27 (IBM Corp., Armonk, NY, USA). Descriptive statistics such as frequencies, percentages, averages, and standard deviations were used to create demographic and occupational data for the participants. Both procedural and statistical methods were used to reduce and evaluate common method bias since all data were gathered using self‐administered questionnaires at a single time point. To reduce evaluation apprehension and social desirability bias, participants were told that their answers would remain anonymous and confidential, that participation was optional, and that there were no right or wrong answers. In addition, statistically, Harman’s single‐factor test was carried out by concurrently running all measurement items through an exploratory factor analysis without rotation. The percentage of total variation explained by the first unrotated factor was investigated as a measure of potential common method bias. The first unrotated factor accounted for 40.081% of the total variance, which was less than the standard 50% threshold. These findings revealed that a single factor did not account for the majority of the covariance among the measured variables, indicating that there was no significant common method bias based on the test [37]. Prior to conducting inferential statistical modeling, rigorous data cleaning procedures were employed. The dataset was systematically screened for missing values, out‐of‐range responses, and outliers to ensure data accuracy. Correlations between the research variables were evaluated using Pearson’s correlation coefficients. To ascertain the mediating role of resilience and psychological empowerment on the relationship between professional commitment and intention to leave, a path analysis of observed variables was conducted using AMOS 24.0 software using the bootstrap approach (5000 repetitions, 95% bias‐corrected confidence interval). The analysis used the validated total/composite scores specified by the original scoring procedures of the instruments. Specifically, total psychological empowerment, total intention to leave, total nursing professional commitment, and total resilience scores were entered as observed variables. Specifically, total psychological empowerment, total intention to leave, total nursing professional commitment, and total resilience scores were entered as observed variables. Aggregation to total scores was considered appropriate because the present study focused on the overall constructs and their hypothesized direct and indirect relationships, rather than on relationships among individual dimensions or subscales. Although psychological empowerment, intention to leave, and nursing professional commitment comprise multiple theoretically defined dimensions, the present study focused on the overall constructs rather than the individual dimensions; therefore, their established total scores were retained as the unit of analysis. This approach was consistent with the primary objective of testing the hypothesized direct and indirect relationships among the global study constructs while avoiding unnecessary model complexity associated with estimating multiple first‐order factors and their indicators.

The use of composite scores was further supported by the established psychometric properties of the instruments and their satisfactory internal consistency in the present sample, with Cronbach’s alpha coefficients ranging from 0.90 to 0.98. Accordingly, the analysis was specified as an observed‐variable path model rather than a latent‐variable structural equation model. A confirmatory factor analysis (CFA) measurement model was therefore not estimated. The goodness‐of‐fit for the structural path model was evaluated using established threshold criteria: a ratio of chi‐square to degrees of freedom (CMIN/DF) of less than 3.0, Tucker–Lewis Index (TLI), goodness‐of‐fit index (GFI), comparative fit index (CFI), and normed fit index (NFI) values greater than 0.90, and a root mean square error of approximation (RMSEA) of less than 0.08. The obtained indices (CMIN/DF = 2.994, TLI = 0.956, GFI = 0.997, CFI = 0.993, NFI = 0.989, and RMSEA = 0.068) demonstrated an excellent fit to the data, with significance determined at a *p* value < 0.05 [38].

## 5. Results

As shown in Table 1, the mean age of participants was 25.97 ± 1.91, and around two‐thirds (63.5%) were female. The majority of participants (88.8%) held a bachelor’s degree, worked in the general ICU (59.1%), had outside work (61.6%), and had experience–2–5 years in ICUs (54.4%). In addition, more than half (51.9%) worked at a nurse‐to‐patient ratio of 1:2.

**TABLE 1:** Sociodemographic characteristics of the participants (*N* = 430).

Variables	Categories	*n*	%
Age	25.97 ± 1.91		
	≤ 25	168	39.1
	> 25	262	60.9

Sex	Male	157	36.5
	Female	273	63.5

Level of education	Diploma	29	6.7
	Bachelor	381	88.6
	master	20	4.7

Working unit	General ICUs	254	59.1
	Coronary ICUs	95	22.1
	Cardiac ICUs	35	8.1
	neurological ICUs	46	10.7

Experience in ICUs (in years)			
	< 2	119	27.7
	2–5	234	54.4
	> 5	77	17.9

Work outside			
	Yes	265	61.6
	No	165	38.4

Nurse‐to‐patients ratio	1:1	156	36.3
	1:2	223	51.9
	1:3	51	11.9

Abbreviation: ICUs, intensive care units.

As shown in Table 2, participants reported moderate to high levels of psychological empowerment (MPS = 69.40), with the highest scores observed for impact (MPS = 77.33) and competence (MPS = 72.33), while meaning was comparatively lower (MPS = 57.00). The intention to leave was relatively high overall (MPS = 79.22), particularly for perceived alternative work opportunities (MPS = 80.88) and turnover intention (MPS = 80.32). Resilience demonstrated a moderate level (MPS = 61.10), with higher scores for flexibility and lower scores for optimism. Similarly, nursing professional commitment was moderate (MPS = 60.20), with willingness to make an effort scoring higher than maintaining professional membership and belief in goals and values.

**TABLE 2:** Descriptive statistics of the study variables (*N* = 430).

Variables	Mean ± SD	MPS
Meaning (3 items)	13.26 ± 2.09	57.00
Competence (3 items)	16.02 ± 2.68	72.33
Self‐determination (3 items)	15.76 ± 2.87	70.87
Impact (3 items)	16.92 ± 2.88	77.33
**Total psychological empowerment (12 items)**	**61.97 ± 9.90**	**69.40**
Perceived alternative work opportunities (4 items)	16.94 ± 5.18	80.88
Job expectations (4 items)	16.23 ± 4.68	76.43
Turnover intention (4 items)	16.85 ± 4.57	80.32
**Total intention to leave (12 items)**	**50.03 ± 14.21**	**79.22**
Flexibility (2 items)	5.42 ± 1.68	67.74
Self‐efficacy (3 items)	7.22 ± 2.48	60.15
Optimism (3 items)	6.63 ± 2.55	55.24
Ability to regulate emotion (1 item)	2.57 ±0 .93	64.24
Cognitive focus/maintaining attention under stress (1 item)	2.59 ± 1.05	64.74
**Resilience (10 items)**	**24.44 ± 8.24**	**61.10**
Willingness to make an effort (13 items)	37.44 ± 10.08	62.65
Maintaining professional membership (8 items)	21.83 ± 6.43	57.61
Having a belief in goals and values (5 items)	13.69 ± 4.06	57.92
**Total nursing professional commitment (26 items)**	**72.96 ± 19.83**	**60.20**

Abbreviations: SD, standard deviation; MPS, mean percentage score.

There was a positive correlation between professional commitment and psychological empowerment (*r* = 0.462, *p* < 0.001) and resilience (*r* = 0.209, *p* < 0.001) while it was negatively correlated with intention to leave (*r* = −0.419, *p* < 0.001). In addition, there was a significant positive correlation between psychological empowerment and resilience (*r* = 0.168, *p* < 0.001) and a negative correlation between psychological empowerment and intention to leave (*r* = −0.290, *p* < 0.001). Finally, a significant negative correlation existed between resilience and intention to leave (*r* = −0.421, *p* < 0.001) (Table 3).

**TABLE 3:** Correlation between study variables.

Variables		1	2	3	4
1. Nursing professional commitment	*r*	1			
	*p*				
2. Psychological empowerment	*r*	0.462^∗∗^	1		
	*p*	< 0.001			
3. Resilience	*r*	0.208^∗∗^	0.168^∗∗^	1	
	*p*	< 0.001	< 0.001		
4. Intention to leave	*r*	−0.419^∗∗^	−0.290^∗∗^	−0.421^∗∗^	1
	*p*	< 0.001	< 0.001	< 0.001	

^∗∗^Correlation is significant at the 0.01 level (2‐tailed).

Using AMOS 24.0, a path analysis model was established with intention to leave as the dependent variable, resilience and psychological empowerment as the mediating variables, and professional commitment as the independent variable. Path analysis was conducted using 5,000 bootstrap samples with 95% bias‐corrected confidence intervals. The adequacy of the measurement model is strongly supported by its goodness‐of‐fit indices: CMIN/DF = 2.994, TLI = 0.956, GFI = 0.997, CFI = 0.993, NFI = 0.989, and RESMA = 0.068. The model is illustrated in Figure 1.

**FIGURE 1:**
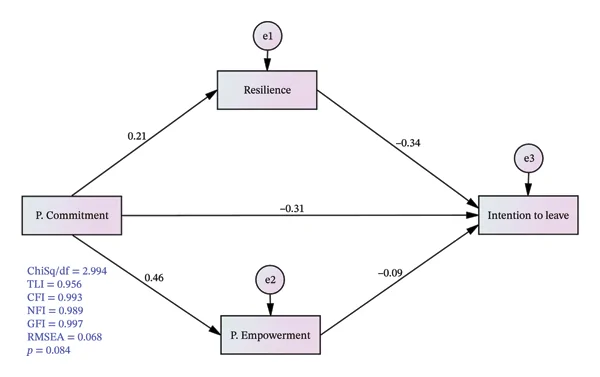
The analysis of the effect of resilience and psychological empowerment on the relationship between professional commitment and intention to leave. Abbreviations: P. commitment, professional commitment; P. empowerment, psychological empowerment.

As presented in Table 4, professional commitment demonstrated a significant total negative effect on intention to leave (*β* = −0.420, 95% CI: −0.478 to −0.357, *p* = 0.005). The indirect effect of professional commitment on intention to leave through resilience was *β* = −0.071 (95% CI: −0.168 to −0.097, *p* = 0.011), while the indirect effect through psychological empowerment was *β* = −0.041 (95% CI: −0.078 to −0.029, *p* = 0.004). The total indirect effect was *β* = −0.112 (95% CI: −0.160 to −0.081, *p* = 0.004), accounting for 26.67% of the total effect. Specifically, the mediating pathway through resilience accounted for 16.90% of the total effect, while the pathway through psychological empowerment accounted for 9.76%. Professional commitment was positively associated with resilience (*β* = 0.208, 95% CI: 0.138 to 0.289, *p* = 0.009) and psychological empowerment (*β* = 0.462, 95% CI: 0.385 to 0.542, *p* = 0.009). Resilience was negatively associated with intention to leave (*β* = −0.343, 95% CI: −0.414 to −0.267, *p* = 0.011), as was psychological empowerment (*β* = −0.091, 95% CI: −0.159 to −0.022, *p* = 0.022). The direct effect of professional commitment on intention to leave remained significant (*β* = −0.306, 95% CI: −0.384 to −0.234, *p* = 0.005), indicating partial mediation.

**TABLE 4:** Path analysis of the mediating effect of resilience and psychological empowerment on the relationship between professional commitment and intention to leave.

Path	Effect size	CI (95%)	*p* value	Proportion mediated (%)
*Indirect effect*				
Professional commitment ‐ > resilience ‐ > intention to leave	−0.071	−0.168 to −0.097	0.011	16.90
Professional commitment ‐ > psychological empowerment ‐ > intention to leave	−0.041	−0.078 to −0.029	0.004	9.76
Total indirect effect	0.112	−0.160 to −0.081	0.004	26.67

*Direct effect*				
Professional commitment ‐ > resilience	0.208	0.138 to 0.289	0.009	
Resilience ‐ > intention to leave	−0.343	−0.414to −0.267	0.011	
Professional commitment ‐ > empowerment	0.462	0.385 to 0.542	0.009	
Psychological empowerment ‐ > intention to leave	−0.091	−0.159 to −0.022	0.022	
Professional commitment ‐ > intention to leave	−0.306	−0.384 to −0.234	0.005	

*Total effect*				
Professional commitment ‐ > intention to leave	−0.420	−0.478 to −0.357	0.005	

Abbreviation: CI, confidence interval.

## 6. Discussion

The present study contributes to the modern nurse management literature by establishing that professional commitment has been associated with intention to leave both directly and indirectly via the parallel mediating roles of psychological resilience and psychological empowerment. Previous research has found that leadership qualities, team culture, autonomous motivation, occupational commitment, supervisor support, colleague behaviors, and nurses’ quality of life are all connected with turnover intention [25–27]. However, most studies focused on direct linkages or diverse organizational antecedents of turnover intention rather than the psychological mechanisms by which professional commitment leads to workforce retention.

Overall, the findings of the present study provide a comprehensive understanding of how professional commitment, psychological empowerment, resilience, and intention to leave are interrelated among CCNs. Professional commitment emerged as the foundational antecedent of the proposed model, demonstrating both a direct negative association with intention to leave and indirect associations through psychological empowerment and resilience. As a result, our findings add new evidence by combining professional commitment, psychological empowerment, and resilience into a single mediation model, providing a more comprehensive explanation of how professional commitment can be translated into decreased turnover intention among CCNs. This integrated perspective provides nursing managers with a stronger theoretical foundation for developing multiple retention strategies that promote professional identity, foster empowerment, and build resilience rather than addressing these elements separately.

### 6.1. Psychological Empowerment, Intention to Leave, Resilience, and Nursing Professional Commitment

Participants’ mean scores reflected a relatively high level of commitment. This finding suggests that the nurses included in the study were committed to their profession. This corresponds to the overall tendency that commitment is a vital aspect of nursing that affects the general satisfaction and retention of nurses in the field [39]. Professional commitment is known to enhance productivity and satisfaction and significantly influence retention rates among nurses. Nonetheless, this commitment may be affected by professional and organizational factors, work–life balance, and personal health [39]. Foodani et al. (2024) [39] identified a range of professionally and organizationally oriented factors that lower professional commitment, such as interpersonal relations, personal coping and well‐being strategies, work–life balance, organizational culture, and attributes related to nursing itself, including emotions, values, and ethics [40]. Viewed through the lens of organizational commitment theory, participants’ high mean scores reflect a strong emotional and obligation‐based attachment to the nursing profession. This intrinsic professional commitment serves as the main buffer, showing that even in high‐stress critical care settings, nurses’ intention to leave their jobs naturally declines when they strongly identify with the fundamental ideals of their profession. Guerrero et al. (2017) proposed that an individual’s commitment to a profession impacts turnover intention, work‐related anxiety, and physiological symptoms of distress in new graduate nurses [41]. This finding highlights the importance of professional commitment to alleviating adverse consequences.

The average assessment score for the intention to leave a job demonstrates that nurses consider leaving their roles. This is troublesome because cognitive exit intentions among many employees tend to come with actual withdrawal, which is detrimental to healthcare organizations. As such, this can be a factor in the intention to leave due to the nursing practice environment and in coping with challenges. Ying et al. (2021) examined the effects of the nursing practice environment on resilience and intent to leave a job among CCNs and suggested that both social support and individual coping skills foster such an environment [42]. Moreover, nurses’ psychological empowerment indicates their perception of the controllable work domains. Psychological empowerment is strongly correlated with work–life quality. Empowerment, based on research, is fundamental to nurses’ perceptions of professional activism in the healthcare system. Recently, graduated nurses require structural and psychological empowerment [43], along with factors such as satisfaction with the work environment, strategic career design, and the level of these determinants.

Psychological empowerment and QWL have a strong positive relationship and parallel each other’s characteristics. This has been noted in an earlier study of primary care nurses and midwives in Jordan [44] in which empowerment is not simply an individual phenomenon, but one that connects to the broader context of work‐related well‐being. Conversely, the mean resilience score suggests that nurses had a moderate degree of resilience. Resilience is one of the most important psychological capacities that enables nurses to endure demanding conditions [9, 39]. Joker et al. (2022) highlighted the significance of resilience and professional commitment as key factors that help nurses to try time [45]. Scholars have investigated the mediating role of perceived organizational support in the relationship between psychological resilience and the intention to stay. As a result, there was an illustration of the balance between individual and organizational factors. It can also be linked to the intention to stay, with perceived organizational support acting as a mediator [45].

### 6.2. Correlation Between Professional Commitment and Psychological Empowerment and Resilience and Intention to Leave

Professional commitment positively correlated with psychological empowerment and resilience. This means that nurses with higher professional commitment tended to experience greater psychological empowerment and exhibit greater resilience. The relationship between commitment and empowerment is well documented. For example, Al‐Obiedat et al. (2024) showed a positive relationship between psychological empowerment and quality of work life, which is a component of commitment among nurses in Jordan [44]. Joker et al. (2022) also highlighted the focus on resilience and professional commitment, noting that these features assist nurses in managing difficult situations [45]. Psychological empowerment positively correlated with resilience, indicating that more empowered nurses exhibited greater resilience. This underscored the role of empowerment in nurses’ professional development and job satisfaction, which can indirectly enhance resilience [46]. The need to cultivate an empowering organizational culture strongly supports the enabling role of psychological empowerment in nurturing resilience among nurses.

Resilience is critical to address, especially for younger nurses, in efforts to ease their psychological strain [47]. Empowerment strategies, when effective, not only relieve stress but also improve coping skills, which, in turn, create a cycle of increased resilience and empowered individuals [48]. It has been demonstrated that resilient individuals manage work‐related stress and the extent to which empowered nurses perceive enhanced psychosocial well‐being and greater capacity to withstand professional challenges [48]. This further reinforces the need to cultivate an environment that psychologically empowers nurses to enhance resilience, which is essential for quality patient care. Moreover, the current results show a considerably negative relationship between professional commitment and intention to leave, which suggests that nurses who are more professionally committed are less likely to leave their jobs. This supports Guerrero et al.’s (2017) findings, which link professional commitment to turnover intention [41].

Similarly, psychological empowerment showed a negative relationship with intention to leave, indicating that empowered nurses did not leave as easily. This is consistent with research demonstrating that factors associated with empowerment impact job satisfaction and quality of work life, thereby decreasing the propensity to leave [41]. Resilience also showed a negative relationship with intention to leave, meaning that more resilient nurses tended to have a lower intention to leave. Ying et al. (2021) supported this finding by examining it among CCNs [41], whereas Pu et al. (2024) discussed the relevance of resilience to nurses’ intentions to stay [13]. These negative correlations provide substantial evidence for the COR theory. In the challenging environment of critical care, resilience and psychological empowerment serve as crucial personal resources. Empowered and resilient nurses are better able to recharge their psychological reservoirs, minimizing the emotional depletion that often drives the desire to leave. Overall, these correlations highlight the extent to which professional commitment, psychological empowerment, and resilience intertwine with retention issues in critical care nursing [49]. Strengthening one’s professional commitment, along with psychological empowerment and resilience, may be critical strategies for reducing the intention to leave and, therefore, stabilizing the workforce.

### 6.3. Effect of Resilience and Psychological Empowerment on the Relationship Between Professional Commitment and Intention to Leave

The path analysis model delineates the relationships between professional dedication, resilience, psychological empowerment, and the intention to quit the organization. The model depicts a sequential protective mechanism, which is consistent with organizational commitment theory and COR theory. Deep professional commitment creates a foundation for professional identity, which allows the accumulation of psychological resources (resilience and empowerment). According to COR theory, nurses who have access to these comprehensive tools are substantially less likely to experience burnout and turnover. The effects both direct and indirect professional commitment on the intention to leave through resilience and psychological empowerment were sustained by the literature. This data point regarding the direct effect of professional commitment on intention to leave supports the evidence identifying professional commitment as one of the primary determinants of employees’ retention or exit decisions. Szoko et al., 2023 [50] argued that higher psychological empowerment is likely to enhance job satisfaction and professional commitment, which, in turn, decreases turnover intentions. Researchers have also argued that employee turnover will decrease as organizational commitment increases due to management’s commitment to psychological safety and empowerment [51].

The mediation effects identified in the model are notable in understanding the importance of resilience and psychological empowerment as constituent factors of employee behavior. An earlier study offers empirical evidence for this pathway, confirming that psychological empowerment significantly mediates the impact of workplace commitment on behavior [52]. Furthermore, it can be argued that effective human resource policies aimed at enhancing psychological empowerment and resilience positively correlate with performance and strengthen their role in retention strategies [53]. Strikingly, the findings seem to diverge from some of the literature describing the impact of transformational leadership on psychological empowerment, job satisfaction, and employee retention. A previous study found that although transformational leadership greatly fosters psychological empowerment, it does not have any direct influence on resilience or employee preparedness to embrace change [54]. This indicates that, while leadership styles may enhance psychological empowerment, the factors that determine employee intentions are intricate and layered.

It can be assumed that developing emotional intelligence will also increase employees’ resilience and decrease their intent to leave. This is in light of findings on the positive relationship between emotional intelligence, resilience, and psychological empowerment among healthcare professionals [24]. This further highlights that the role of emotional intelligence as an additive within the professional commitment–resilience–empowerment triad has yet to be discovered and investigated. Thus, the path analysis provided makes a substantial contribution to existing knowledge by illuminating the intricate dynamics of professional commitment and resilience, psychological empowerment, and intent to leave. The identified negative consequences strengthen employee behavior theories and concede practical strategies for organizational leadership aimed at enhancing employee loyalty and satisfaction. More work is needed in this area, especially regarding the relationships between emotional intelligence, leadership, and these core constructs.

### 6.4. Study Limitations

Our study focused on CCNs’ intention to leave as well as the impact of resilience and psychological empowerment on the relationship between professional commitment and intention to leave. However, the use of self‐reported perception‐based data collected from CCNs who participated in the questionnaires was restricted, leading to self‐reported bias. Future research should combine quantitative and qualitative methods. Another problem was that the study was cross‐sectional, included only public hospitals, and utilized a convenience sampling method, limiting the generalizability of the findings. Because probability sampling was not employed, the assumptions underlying traditional inferential statistics are not fully met, and the results should be interpreted with caution as exploratory. Accordingly, the interpretation of our path model prioritizes the magnitude and precision of the relationships—specifically the standardized effect sizes and the 95% bias‐corrected confidence intervals—rather than relying solely on *p* values and strict statistical significance. Furthermore, the directionality of the proposed model is another limitation. Even though our framework viewed professional dedication as a precursor to resilience and psychological empowerment, which, in turn, influenced the desire to leave, it is crucial to recognize that other theoretical models might also be tenable. Thus, we suggest that to examine alternate and possibly reciprocal routes among these variables, future research should use experimental or longitudinal approaches. Further understanding of the complex interactions between empowerment, resilience, professional commitment, and intention to leave may be possible by investigating such models. Finally, the path analysis was carried out with observed composite scores rather than latent‐variable constructs. While the observed‐variable approach was justified by the instruments’ high internal consistency and established validity, a recognized trade‐off is that measurement error was not explicitly modeled across the various multidimensional subscales. Consequently, the analysis lacks the full measurement‐error correction and latent‐construct estimation benefits of a complete structural equation modeling technique. Nonetheless, the use of known composite scores was deemed adequate for the study’s goal of investigating the hypothesized direct and indirect correlations between the study variables. Future research might use a full latent‐variable SEM with item‐level indicators, CFA, and a thorough examination of convergent and discriminant validity to further examine the suggested model while explicitly accounting for measurement error.

### 6.5. Recommendations or Implications for Practice

The present study demonstrated that professional commitment was a predictor of lower intention to leave, both directly and indirectly through psychological empowerment and resilience. Therefore, healthcare organizations should implement strategies that strengthen nurses’ professional commitment by promoting professional identity, reinforcing ethical nursing values, fostering long‐term career engagement, and providing structured career development pathways, opportunities for specialty certification, mentorship programs for early‐career nurses, and recognition and reward systems.

The findings also revealed that psychological empowerment significantly reduced nurses’ intention to leave. Accordingly, nurse managers should cultivate empowering work environments by encouraging nurses’ participation in clinical and organizational decision‐making, promoting shared governance, delegating appropriate authority, providing regular constructive feedback, recognizing professional achievements, and ensuring access to the resources required for autonomous and competent practice. These interventions can enhance nurses’ perceptions of meaning, competence, self‐determination, and organizational impact.

Furthermore, because resilience mediated the relationship between professional commitment and intention to leave, healthcare institutions should implement resilience‐enhancement initiatives, including stress management training, resilience workshops, peer‐support groups, structured debriefing sessions following stressful clinical events, employee assistance programs, psychological counseling services, and well‐being initiatives that strengthen healthy coping strategies and emotional resilience.

### 6.6. Implications for Nursing Management

The findings of this study indicate that reducing turnover intention among CCNs requires an integrated nursing management approach rather than isolated interventions. Nursing administrators should develop comprehensive retention strategies that simultaneously strengthen professional commitment, psychological empowerment, and resilience through supportive leadership, participatory management, adequate staffing, manageable workloads, healthy work environments, and continuous professional development opportunities.

These findings also suggest that nursing management policies should prioritize the development of organizational cultures that recognize nurses’ professional contributions, promote empowerment and resilience, and support career advancement. By addressing these modifiable organizational and psychological factors simultaneously, healthcare organizations can improve nurse retention, enhance workforce stability, reduce turnover‐related costs, and ultimately ensure the delivery of safe, high‐quality, and continuous patient care in critical care settings.

## 7. Conclusion

Higher nursing professional commitment positively resonates with psychological empowerment and resilience, while negatively correlating with intention to leave. It appears that nurses who commit professionally tend to be more empowered, better able to cope with work challenges, and less likely to seek alternative employment. Positive relationships also exist between psychological empowerment and resilience, with the latter being negatively correlated with intention to leave. As such, it further reinforces the notion that having strong control and the ability to recover from setbacks are vital for retaining nurses. The path analysis clarifies these relationships, explaining that professional commitment, through fostering resilience and psychological empowerment, indirectly lowers turnover intention while directly reducing it. These findings emphasize the need to cultivate professional commitment, psychological empowerment, and resilience in nurses as effective measures to enhance their retention in the nursing workforce.

## Author Contributions

Sameer A. Alkubati: conceptualization, methodology, formal analysis, validation, visualization, writing–original draft, and writing–review and editing; Shimmaa Mohamed Elsayed: conceptualization, methodology, data curation, investigation, writing–original draft, and writing–review and editing; Ibrahim Alasqah: funding acquisition, project administration, supervision, and writing–review and editing; Ahmed Hashem El‐Monshed: conceptualization, methodology, investigation, and writing–review and editing; Heba E. El‐Gazar: data curation, investigation, methodology, and writing–review and editing; Eddieson Pasay‐an: formal analysis and writing–review and editing; Nada Alqarawi, Wesam T. Almagharbeh, Mohamed A. Zoromba, Talal Al‐Qalah, Basma Salameh, Mesheil Alalyani, and Majid Ali Alotni: investigation and writing–review and editing.

## Funding

The Researchers would like to thank the Deanship of Graduate Studies and Scientific Research at Qassim University (https://www.qu.edu.sa) for financial support (QU‐APC‐2026).

## Disclosure

All authors approved the final version of the manuscript and agree to be accountable for all aspects of the work.

## Ethics Statement

Ethical approval for this study was obtained from the Ethics Committee of the Faculty of Medicine, Alexandria University (approval no. 0306882; September 8, 2024). The study was conducted in accordance with the principles of the Declaration of Helsinki. Informed consent was obtained from all participants prior to data collection.

## Conflicts of Interest

The authors declare no conflicts of interest.

## Supporting Information

Additional supporting information can be found online in the Supporting Information section.

## Supporting information


**Supporting Information** The completed Strengthening the Reporting of Observational Studies in Epidemiology (STROBE) checklist for cross‐sectional studies is provided as supporting information to demonstrate adherence to recommended reporting standards.

## Data Availability

The data are available at request from the corresponding author.
